# Protective effects of a mixture of multi-strain native Iranian probiotics on lead acetate-induced toxicity in the kidney of male rats: An integrated biochemical, molecular and histopathological study 

**DOI:** 10.22038/AJP.2024.24261

**Published:** 2024

**Authors:** Mohsen Akbaribazm, Zahra Abdol Al amir Mohammad Javad, Seyed Naser Alavi, Zahra Keshtmand

**Affiliations:** 1 *Department of Basic Medical Sciences, Khoy University of Medical Sciences, Khoy, Iran*; 2 *Department of Biology, Science and Research Branch, Islamic Azad University, Tehran, Iran *; 3 *Department of Biology, Central Tehran Branch, Islamic Azad University, Tehran, Iran *

**Keywords:** Probiotic, Lead acetate, Kidney, Inflammation, Oxidative stress

## Abstract

**Objective:**

In this study, the protective effects of native Iranian probiotics (*Lactobacillus rhamnosus, Lactobacillus casei *and* Lactobacillus holoticus*) on lead acetate (PbAc)-induced toxicity in the kidney of male rats were investigated using biochemical, molecular and histopathological approaches.

**Materials and Methods:**

Twenty-one male Wistar rats were divided into three groups (n=7/group), including controls, PbAc recipient (10 mg/kg) and PbAc recipient (10 mg/kg) + probiotic mixture (10^9^ CFU). PbAc and probiotics were gavage in the groups. On the 31^st^ day, blood samples were used to measure serum concentrations of creatinine (Cr), blood urea nitrogen (BUN), sodium, total protein and potassium. Rats were dissected and renal tissues apoptotic and inflammatory genes were evaluated.

**Results:**

PbAc increased serum concentrations of Cr, sodium, and urea, and decreased total protein and potassium, while it enhanced interleukine-6 (IL-6) and tumor necrosis factor -α (TNF- α) gene expression in kidney tissue compared to the control group. Probiotic mixture decreased Cr, BUN, and malondialdehyde and increased activity of catalase and superoxide dismutase enzymes in kidney tissue.

**Conclusion:**

The results of the study showed that the native Iranian probiotics mixture can be used to protect the function and structure of the kidneys against toxic and oxidative damage induced by PbAc.

## Introduction

Air pollution is one of the biggest current and tangible problems in the world and causes damage to humans and other living beings. Industrial waste, radioactive materials, municipal waste, chemicals and natural pollutants (volcanoes, etc.) are among the polluting sources (Briffa et al., 2020). One of the primary air pollutants is lead (Pb), which does not occur naturally in the atmosphere. Its release into the air constitutes a significant form of pollution with widespread consequences. Exposure to Pb can result in various health problems, such as damage to the glomeruli, degeneration of liver tissue (parenchyma), neurological disorders, epilepsy, anemia, and genetic disorders. (Dolenc et al., 2021).

Lead acetate (PbAc) indirectly produces reactive oxygen species (ROS), which can disturb the internal antioxidant status and damage the lipids of biological membranes and change the transport of substances through the Na^+^ - K^+^ ATPase pump (Annaç et al., 2022). Pb accumulates in the kidney and induces apoptosis in tubular and glomerular cells. In addition, Pb deactivates glutathione by forming a bond with sulfhydryl groups and prevents the synthesis of glutathione (*GSH*) from cysteine through the Y-glutamyl cycle (Yousef et al., 2019). Also, intraperitoneal injection of PbAc increases the peroxidation of unsaturated fatty acids, accumulation of free radicals, and decreases the concentration of copper and zinc, iron, selenium, glutathione, superoxide dismutase (SOD), catalase (CAT), and glutathione peroxidase enzymes in liver and kidney tissues (Yuniarti et al., 2021). 

Probiotics, renowned for their antioxidant and anti-inflammatory properties, have proven effective in scavenging free radicals by augmenting the activity of endogenous antioxidant enzymes and enhancing overall antioxidant capacity (Wang et al., 2017). Moreover, they have been shown to regulate the secretion of cytokines and immunoglobulins by the immune system, and modulate the microbial flora of the digestive system (Cristofori et al., 2021). Numerous studies have elucidated various protective mechanisms of probiotics, including the suppression of pro-inflammatory cytokines secretion in immune and damaged cells. Additionally, some probiotics can inhibit the mitochondrial apoptotic cascade by targeting apoptotic proteins such as Bcl-2, TNF receptor-associated factor 1 (TRAF-1), and survivin. Some probiotics also influence cell cycle-regulating proteins like cyclin D, thereby modulating the cycle of damaged and normal cells, and regulating the proliferation and differentiation of parenchymal cells and stem cells (Liu et al., 2022; Dehghani et al., 2021). In addition, in the study of Barzin Oshtologh et al. (2022), a mixture of these native Iranian probiotics (Lactobacillus rhamnosus, Lactobacillus casei and Lactobacillus holoticus) protected the intestine against inflammatory and oxidative damage of PbAc (Barzin Oshtologh et al., 2022).

With this background in mind, the present study aims to evaluate the protective effects of native Iranian probiotics against PbAc-induced kidney toxicity in male rats using biochemical, molecular, and histopathological approaches. By employing these comprehensive methodologies, we aimed to gain insights into the potential therapeutic benefits of probiotics in mitigating kidney damage.

## Materials and Methods

### Probiotic mixture preparation

Mix-probiotics native to Iran including *Lactobacillus rhamnosus* (IBRC-M11322), *Lactobacillus holoticus* (TG-35) and *Lactobacillus casei* (IBC-M10784) bacteria were purchased in powder form with a concentration of 10^10^ CFU/ml from Techgen Bio Company. Then, 1 ml of probiotics was dissolved in 9 ml of distilled water (DW) (final dose of 1×10^9^ CFU/ml) (Keshtmand et al., 2021). 

### Animals and experimental design

According to Table 1, a total of twenty-one male Wistar rats weighing 180±20 grams each were included in this study and divided into 3 groups (n=7/group). Prior to the start of the study, a 3-day adaptation period was provided to allow the rats to acclimate to their surroundings and cages. The rats were maintained under standardized conditions at a temperature of 25±3°C, a humidity level of 40±3%, and a 12/12 light/dark cycle. Throughout the study, the rats had unrestricted access to drinking water and they were fed with standard laboratory animal pellets. All procedures adhered to international standard guidelines and protocols, with approval obtained from the research ethics committees of Islamic Azad University, Central Tehran Branch (approval number: IR.IAU.CTB.1400.027).

Group 1 (Control group): Rats received a daily oral gavage of 0.5 ml (500 µl) of distilled water (DW) for 30 days.

 Group 2 (PbAc group (PbAc)): Rats received intraperitoneal injections of 10 mg/kg PbAc dissolved in 500 µl of DW on days 1, 2, and 3 of the study.

Group 3 (PbAc and 10^9^ CFU probiotic mixture treatment groups (PbAc + probiotics)): Rats received intraperitoneal injections of 10 mg/kg PbAc dissolved in 500 µl of DW on days 1, 2, and 3 of the study, along with an oral gavage of 10^9^ CFU probiotic mixture for 30 days. The probiotic mixture consisted of *L. rhamnosus*, *L. holoticus *and *L. casei*.

During the study, PbAc was administered on days 1, 2, and 3, while the probiotic mixture was administered consecutively for 30 days. The administration of PbAc and the probiotic mixture occurred at specific times of the day (9 am and 3 pm, respectively). The optimal dosage, effective yet non-toxic, was determined based on the LD_50_, pilot study, and previous research (Dashtbanei and Keshtmand, 2023; Akbaribazm et al., 2021).

### Kidney serum related biochemical parameters

On day 31, anesthesia was induced using ketamine (80 mg/kg) and xylazine (10 mg/kg). Blood samples were then collected from the heart to assess the levels of serum biochemical factors associated with the kidneys. The levels of BUN, Cr, Na^+^, and K^+^ were determined using an autoanalyzer (ChemWell T-4620 biochemistry autoanalyzer, Tehran, Iran). Additionally, the measurement of total protein (TP) was performed using the Pars Azmoon ELISA kit (catalog number: PA37001-300; Pars Azmoon, Tehran, Iran), following the manufacturer's instructions (Akbaribazm et al., 2021).

### Nitric oxide (NO) assay

The Griess method was employed in this study to quantify the levels of nitric oxide (NO) in serum samples. The procedure involved combining 500 μl of serum samples, 6 mg of zinc oxide, and 500 μl of Griess solution. The mixture was centrifuged at 10,000 g for 15 min and subsequently incubated at 37°C for 60 min. After the incubation period, the resulting mixture was evaluated for absorbance at 540 nm and 630 nm using a UV-1280 Shimadzu spectrophotometer (Shimadzu, Japan) (Akbaribazm et al., 2021).

### Kidney tissue activity of catalase (CAT)

The kidney tissue CAT activity was evaluated using the ZellBio ELISA-based commercial kit according to the instructions and recommendations of the manufacturer (catalog number: ZB-CAT-48A; ZellBio GmbH, Germany). The absorbance of the samples was measured at a wavelength of 405 nm using a Stat Fax ELISA reader (model number: 303, United States). The CAT enzyme activity was determined using the recorded absorbance values and the provided formula.

Catalase activity (v/ml) = (OD _blank_ - OD _sample_) × 271 × (1/60 × sample volume) (Oliaei et al., 2023). 

### Kidney tissue activity of superoxide dismutase (SOD)

The kidney tissue SOD activity was determined using the Pars Azmoon ELISA-based commercial kit according to the instructions and recommendations of the manufacturer (catalog number: PASOD-96; Pars Azmoon, Tehran, Iran). The optical absorption of the samples was measured at a wavelength of 405 nm using a Stat Fax ELISA reader (model number: 303, United States), and the recorded value was referred to as the OD of the sample. Finally, the SOD enzyme activity was measured using the following formula.

SOD activity (U/ml or mg protein) = (OD _Test_/OD _control_) × 200 (Oliaei et al., 2023). 

### Kidney tissue malondialdehyde (MDA) levels

The kidney tissue MDA levels were assessed using the ZellBio ELISA-based commercial kit, according to the instructions and recommendations of the manufacturer (catalog number: ZX-44116-96; ZellBio GmbH, Germany). The absorbance of the supernatant was measured at a wavelength of 535 nm. The tissue MDA amount was determined based on the standard curve (Oliaei et al., 2023).

### Kidney tissue TNF-α and IL-6 genes expression

Total RNA was extracted from kidney tissue using EX6101-RNX Plus Solution (catalog number: EX6101-RNX; SinaClon BioScience, China). Briefly, 50 gram of kidney tissue were lysed using RNX Plus buffer, followed by the addition of 200 μl of chloroform. The samples were then centrifuged at 13,000 g for 15 min at 4°C. The resulting supernatant was mixed with cold isopropanol (200 μl) and incubated on ice for 15 min, followed by centrifugation at 13,000 g for 15 min. Afterward, 1 ml of 50% ethanol was added to the samples, and centrifuged for 15 min at 13,000 g at 4°C. Finally, the remaining pellet was suspended in 50 μl of DW and stored at -80°C.

The Revert AidTM First Strand cDNA Synthesis Kit (catalog number: K1621; Fermentas, USA) was employed for cDNA synthesis following the manufacturer's instructions. The sequence of TNF-α and IL-6 genes was designed using gene runner software (Hastings Software, Hudson, United States). After designing, the genes were subjected to a blast search in the NCBI database (https://www.ncbi.nlm.nih.gov/tools/primer-blast/) for validation. The housekeeping gene, β-actin, was used as a reference gene (Table 2). Amplification of the genes was performed using forward and reverse primers with 42 temperature cycles on a Corbett Rotor thermocycler. Each temperature cycle consisted of 5 min at 70°C as annealing temperature. The relative expression of the genes was determined using the threshold cycle (Ct) of each gene, ∆∆Ct, and the fold change formula.

∆∆Ct = [(Ct_ sample__-_ Ct _housekeeping gene_) - (Ct _sample_ – Ct _control_)]. 

fold change = 2 ^-∆∆CT^ (Akbaribazm et al., 2020). 

### Kidney tissue histopathology assay

On the 31^st^ day of the study, kidney samples were extracted from the surrounding tissues. These samples were then fixed in 10% formalin for 72 hr and subsequently processed into paraffin blocks using L-shaped molds. From these blocks, 5-μm sections were obtained and underwent routine histological procedures. The tissue sections were stained with periodic acid-Schiff (PAS) and Jones' Methenamine Silver stains. Finally, the slides were examined under an optical microscope (Olympus IX71 microscope) connected to a Kecam Technologies camera at a magnification of 100× and 400× (Akbaribazm et al., 2020). 

### Statistical analysis

The graphs were designed using GraphPad Prism 8 software, while the statistical analysis of the study was performed using SPSS-16 software. The results were analyzed using one-way ANOVA followed by Tukey's post-hoc test. The data are presented as mean±standard deviation (SD), and a p<0.05 was considered statistically significant.

## Results

### Kidney serum related biochemical parameters

#### Serum BUN and Cr levels

**Figure 1:**
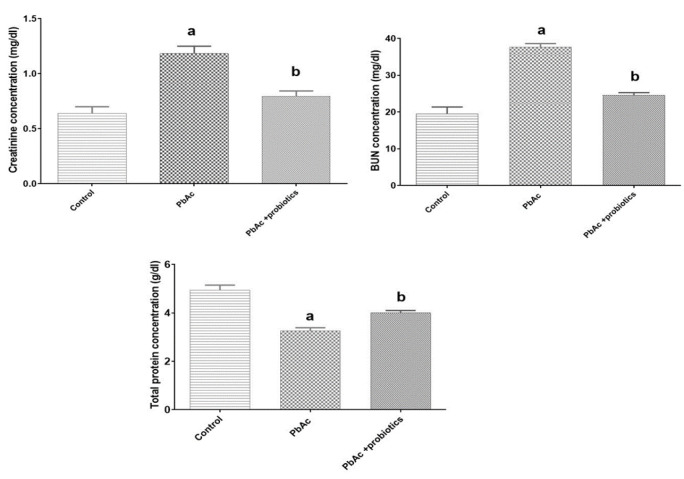
Serum levels of creatinine (µmol/l), blood urea nitrogen (BUN) (mg/dl), and total protein (g/dl) in experimental groups (means±SD). ^a ^(p<0.05) control *vs.* PbAc groups and ^b^ (p<0.05) PbAc *vs.* probiotic mixture treated groups.

The assessment of biochemical parameters related to kidney function revealed significant (p<0.05) increases in BUN and Cr levels, as well as a significant (p<0.05) decrease in TP levels in response to PbAc administration compared to the control group. However, treatment with probiotics (PbAc + probiotic group) resulted in significant (p<0.05) decreases in BUN and Cr levels and significant (p<0.05) increases in TP levels compared to the PbAc group (Figure 1).

#### Serum Na+ and K+ ions levels

**Figure 2:**
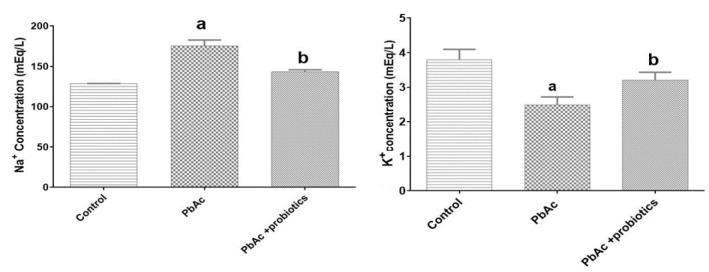
Serum levels of Na^+^ (mEq/l) and K^+^ (mEq/l) in experimental groups (means±SD). ^a ^(p<0.05) control *vs.* PbAc groups and ^b^ (p<0.05) PbAc *vs.* probiotic mixture treated groups.

The assessment of serum levels of Na^+^ and K^+^ ions revealed that PbAc administration resulted in a significant (p<0.05) increase in serum Na^+ ^levels and a significant (p<0.05) decrease in serum K^+ ^levels compared to the control group. These alterations were attributed to impaired glomerular filtration function. However, treatment with probiotics (PbAc + probiotic group) restored the Na^+^- K^+ ^ionic balance. Specifically, compared to the PbAc group, the probiotic treatment led to a significant (p<0.05) decrease in serum Na^+ ^levels and a significant (p<0.05) increase in serum K^+ ^levels (Figure 2). 

#### Kidney tissue MDA levels and CAT and SOD activities

**Figure 3:**
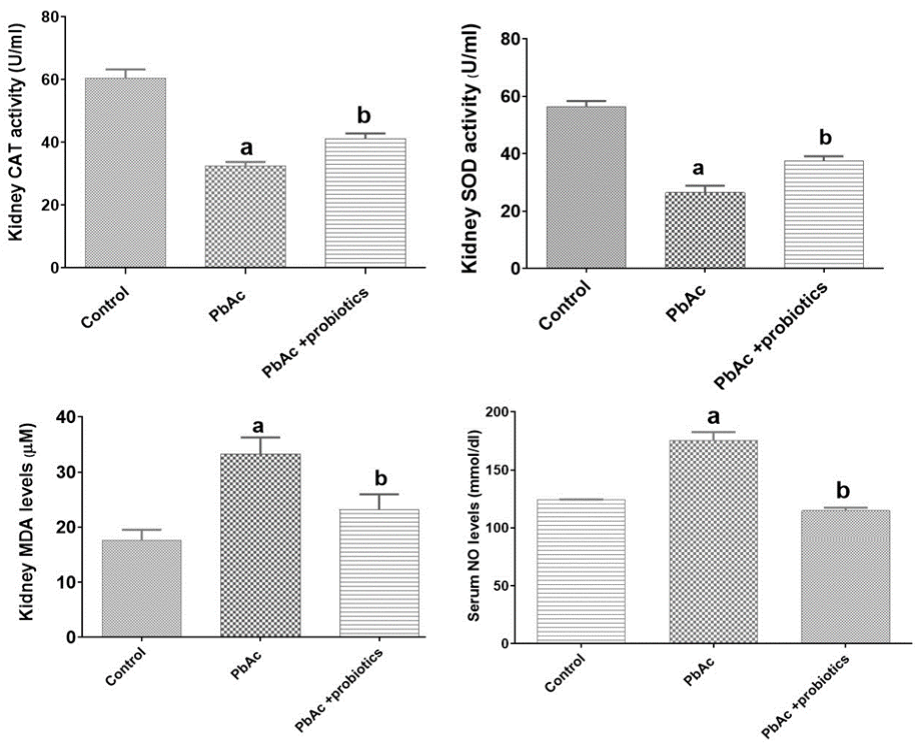
Serum levels of nitric oxide (NO) (mmol/ml) alongside kidney activity of superoxide dismutase (SOD) and catalase (CAT) (U/ml) and MDA levels (µM/g) in experimental groups (means±SD). ^a ^(p<0.05) control *vs.* PbAc groups and ^b^ (p<0.05) PbAc *vs.* probiotic mixture treated groups.

The administration of PbAc induced the production of free radicals, leading to a significant (p<0.05) decrease in the activity of CAT and SOD enzymes compared to the control group. Consequently, there was a notable increase in lipid peroxidation, as evidenced by the significant (p<0.05) elevation of MDA and NO levels. However, treatment with probiotics (PbAc + probiotic group) demonstrated antioxidant effects, resulting in a significant (p<0.05) increase in the activity of CAT and SOD enzymes compared to the PbAc group. Furthermore, the tissue level of MDA and serum levels of NO exhibited a significant (p<0.05) decrease compared to the PbAc group (Figure 3). 

### Expression of IL-6 and TNF-α in kidney tissue

**Figure 4:**
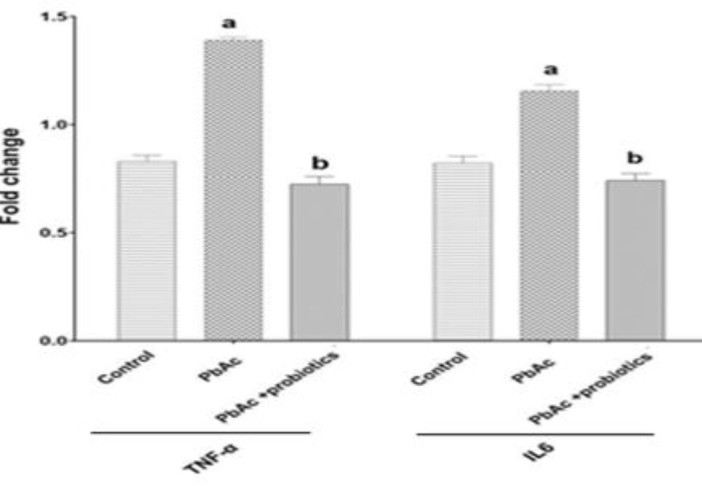
IL-6 and TNF-α gene expression in kidney tissue (means±SD). ^a ^(p<0.05) control *vs.* PbAc groups and ^b^ (p<0.05) PbAc *vs.* probiotic mixture treated groups.

PbAc administration resulted in a significant (p<0.05) increase in the expression of IL-6 and TNF-α in kidney tissue compared to the control group. On the other hand, treatment with probiotics (PbAc + probiotic group) led to a significant (p<0.05) decrease in the expression of IL-6 and TNF-α in kidney tissue (Figure 4).

#### Histopathology of kidney

**Figure 5:**
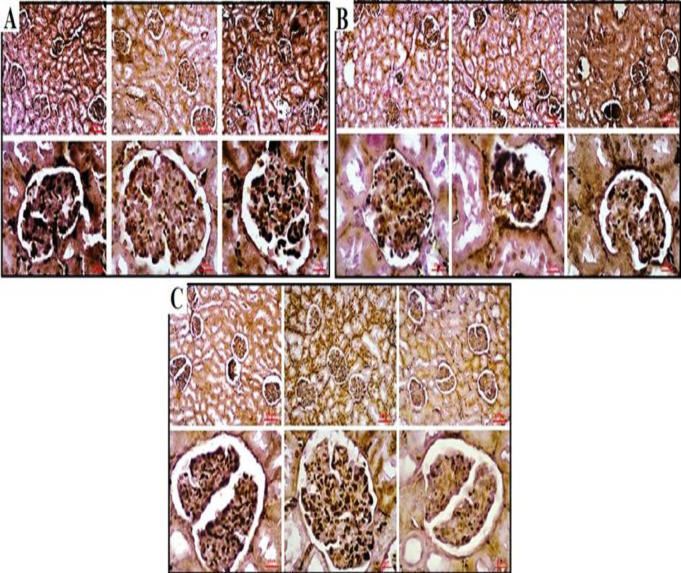
Histopathological findings in kidney tissues in experimental groups [A-C (Jones' Methenamine Silver ×100, scale bar = 100 μm and ×400, scale bar = 25 μm).

**Figure 6:**
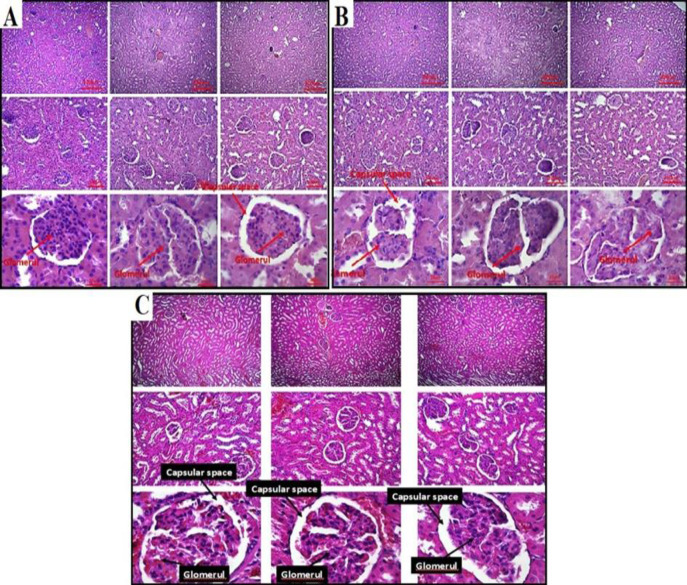
Histopathological findings in kidney tissues in experimental groups [A-C (PAS ×100, scale bar = 100 μm and ×400, scale bar = 25 μm).

Upon histopathological evaluation of the kidney tissue in the PbAc group, several notable findings were observed compared to the control group. These included a significant increase in lymphocyte infiltration, loss of integrity in the tubular and glomerular membranes, and evident shrinkage of tubules and glomeruli. The interstitial tissue volume increased, replacing the tubular parenchyma. Numerous degenerated tubules and glomeruli were observed in the parenchymal space, while the urinary space within the glomeruli decreased. Additionally, apoptotic glomerular cells were present in this space. Renal vessels exhibited hypertrophy, along with signs of hyperemia and congestion. In contrast, the group treated with probiotics (PbAc + probiotic group) showed distinct improvements compared to the PbAc group. Lymphocytic infiltration and degeneration were significantly reduced, and the tubular and glomerular parenchymal tissues exhibited a normal structure and no shrinkage was observed in the tubules or glomeruli in PbAc + probiotic group. In this group, the urinary space within the glomeruli increased, and no apoptotic cells were detected in this area. Evaluation of the vessels in the cortex and medulla of kidney in PbAc + probiotic group revealed the absence of vascular hypertrophy, hyperemia, and congestion (Figure 5 and 6).

## Discussion

The findings of this study highlight the effective role of probiotics in protecting the structure and function of kidney tissue against damage caused by PbAc. This protection is achieved through the probiotics' antioxidant and anti-inflammatory mechanisms.

**Table 1:** Experimental design and animal grouping

**Group number**	**Group name**	**Treatment (dose and prescription manner)**	**Treatment period**
1	Control	500 µl of D.W./ p.o.	30 days
2	PbAc	10 mg/kg lead acetate (PbAc) dissolved in 500 µl of D.W./ i.p.	Days 1, 2, and 3
3	PbAc + probiotic mixture	PbAc +10^9^ CFU probiotic mixture/ p.o.	30 days

PbAc: lead acetate, p.o.: per oral, i.p.: intraperitoneally, and p.d.: per day

**Table 2:** Primer sequences

**Gene**	** Sequences**	**Annealing temperature (°C) **
β-actin	Forward (5′–3′): AAGATCCTGACCGAGCGTGG Reverse (3′– 5′): CAGCACTGTGTTGGCATAGAGG	70
TNF-α	Forward (5′–3′): ACTGAACTTCGGGGTGATTG Reverse (3′– 5′): GCTTGGTGGTTTGCTACGAC	70
IL-6	Forward (5′–3′): TGATGGATGCTTCCAAACTG Reverse (3′– 5′): GAGCATTGGAAGTTGGGGTA	70

### PbAc induced kidney toxicity 

The findings from various studies indicate that Pb exposure leads to an increase in lipid peroxidation, resulting from elevated levels of free radicals in parenchymal cells across different tissues (Matović et al., 2015; Patra et al., 2011). Moreover, Pb inhibits endogenous antioxidant enzymes and directly damages the microvilli of proximal convoluted tubules, leading to tubule degeneration and disruption of plasma-urine ionic balance. Intracellular apoptotic vesicles are also induced as a result of Pb exposure (Kucukler et al., 2021). Additionally, Pb indirectly affects transport activity by altering membrane fluidity through binding to membrane phospholipids and increasing lipid peroxidation (Scholz et al., 2021). In a study conducted by Wahyuningsih et al. in 2020, the toxic effects of Pb acetate on mouse kidneys were investigated. The results revealed that Pb increased BUN and Cr levels. Furthermore, a decrease in glutathione levels and an increase in MDA indicated the presence of oxidative stress in kidney tissues following PbAc exposure (Wahyuningsih et al., 2020). Other studies have reported a significant increase in BUN, Cr, and bilirubin levels in the serum of treated mice compared to the control group in response to heavy metals administration (Salvador et al., 2023). These findings align with the results of the present study, as PbAc elevated BUN and Cr levels through oxidative damage and inflammatory pathways, disrupting the regulatory balance of Na+ and K+ ions. Previous investigations have shown that different concentrations of PbAc disturb kidney function and lead to changes in related blood biochemical parameters. Serum and plasma Cr and BUN concentrations are commonly used as factors to evaluate glomerular kidney function. Moreover, changes in Cr clearance can also reflect alterations in tubule function since Cr is not only filtered but also secreted in the tubules (Kucukler et al., 2021). Andjelkovic et al.'s study in 2019 demonstrated that PbAc increased BUN levels while decreasing urinary urea levels and increasing protein excretion, indicating impaired glomerular function and supporting the observed changes (Andjelkovic et al., 2019). Regarding the evaluation of antioxidant enzymes in kidney tissue, specifically MDA, NO, CAT, and SOD levels, the present study revealed a decrease in CAT and SOD enzyme activity in the PbAc group.

In addition, this study aimed to investigate the expression of inflammatory factors TNF-α and IL-6. The findings revealed a noteworthy upregulation of these genes in the groups administered with PbAc compared to the control group. However, in the group receiving PbAc in combination with a blend of native Iranian probiotics, a considerable reduction in gene expression of TNF-α and IL-6 was observed compared to the PbAc group. Previous studies have consistently reported that PbAc activates diverse inflammatory pathways, resulting in elevated serum levels and increased expression of pro-inflammatory genes. For instance, Kumawat et al. (2014) investigated the toxic effects of PbAc on neurons in the central nervous system and found that this compound induces the production of pro-inflammatory cytokines such as IL-6, TNF-α, cyclooxygenase-2 (COX-2), and monocyte chemoattractant protein-1 (MCP-1) (Kumawat et al., 2014). Similarly, in a study by Bhattacharjee et al. (2021), it was demonstrated that PbAc administration increased the expression of pro-inflammatory genes IL-6, COX-2, and IL-1β in the tissues of all rats exposed to this heavy metal (Bhattacharjee et al., 2021). These findings support the notion that PbAc exposure promotes the upregulation of pro-inflammatory genes, which can be mitigated by the administration of native Iranian probiotics in combination with PbAc.

### Protective effects of Iranian probiotics against PbAc -induced kidney injury

Several animal studies conducted on probiotic mixtures in dogs and cats have demonstrated their ability to regulate the renal excretion of BUN and Cr while maintaining the balance of plasma-urinary levels in these animals. In a rat model of chronic kidney disease, Inatomi and Honma (2023) showed that a time-dependent administration of a probiotic mixture containing *Bacillus subtilis*, *Enterococcus faecium*, and *Clostridium butyricum* regulated the balance of BUN and Cr and preserved kidney structure in adenine-induced renal-damaged rats (Inatomi and Honma, 2023). Probiotics contribute to maintaining the plasma-urinary Na+/K+ balance by clearing urotoxins and regulating urine acid-base balance, including pH levels. Bouhafs et al. (2015) demonstrated the protective effects of a probiotic mixture containing *Lactobacillus plantarum* in pregnant rats exposed to endosulfan. The probiotic mixture inhibited the apoptotic cascade, increased the activity of antioxidant enzymes CAT and SOD, and enhanced the overall antioxidant capacity. Additionally, it protected the urinary parenchyma (tubular and glomerular), preserved kidney function, and reduced serum levels of BUN and Cr (Bouhafs et al., 2015). Furthermore, studies have revealed that probiotic mixtures can inhibit ROS-mediated MAPKs and TLR4/NF-κB pathways, thereby regulating the cell cycle in kidney tissue (Wei et al., 2021). Probiotics also affect different cell cycle regulatory pathways in the G1 and G2/M phases through the Notch/AKT/mTOR and PI3K/AKT/mTOR pathways (Allam et al., 2021).

Probiotics possess the ability to regulate the expression of genes and proteins, particularly aquaporin-2 channels (AQP2), which play a crucial role in maintaining urine and blood osmolality (Michałek et al., 2016). Furthermore, a previous study have indicated that probiotics can regulate the activity of the Na+/K+ ATPase enzyme involved in secretion/reabsorption processes in the convoluted tubules near the kidney and the brush border of tubular cells within these tubules (Yuan et al., 2023). Dashtbanei et al. (2023) demonstrated that specific gut microorganisms, including *Lactobacillus rhamnosus*, *Lactobacillus casei*, and *Lactobacillus holoticus*, not only strengthen the antioxidant system by increasing the activity of SOD and CAT enzymes but also exhibit anti-inflammatory effects by inhibiting TNF-α and IL-6. These probiotics also protect against oxidative injuries induced by cadmium in the small intestine and lung of rats by inhibiting mitochondrial apoptosis pathways, including the downregulation of genes such as Bax, p53, and caspase 3 (Dashtbanei and Keshtmand, 2023). Probiotics influence the acid-alkaline balance in blood and urine through their effects on the activity of loop of Henle cells and urinary collecting ducts, leading to the regulation of levels of K^+^, Na^+^, HCO_3_^-^, and H^+ ^(Kiran et al., 2019). In the present study, native Iranian probiotics (*L. rhamnosus*, *L. casei*, and *L. holoticus*) were found to increase the antioxidant capacity by enhancing the activities of CAT and SOD in the serum and kidney, thereby reducing oxidative stress indicators such as NO and MDA. Additionally, this probiotic mixture exhibited anti-inflammatory effects by suppressing the expression of pro-inflammatory genes TNF-α and IL-6, ultimately contribute to the maintenance of kidney function, regulation of serum-urinary Na+-K+ levels, and the excretion of BUN and Cr. Although the findings of this study demonstrate the potential of native Iranian probiotics in protecting kidney function and structure against oxidative damage induced by PbAc, further investigations, including clinical trials, are necessary to explore additional pathways and potential benefits.

Microbial modulation therapies, including probiotics, hold promise in mitigating renal dysfunction at different stages of chronic kidney disease (CKD). Probiotics have demonstrated immune-modulating properties that can effectively slow down the progression of CKD. This study focused on investigating the anti-inflammatory and antioxidant effects of native Iranian probiotics, specifically *L. rhamnosus*, *L. casei*, and *L. holoticus*, in controlling CKD development induced by Pb toxicity. The results indicate the potential of this probiotic mixture in the treatment of kidney disorders, especially for protecting the kidneys of individuals exposed to heavy metals, considering its cost-effectiveness and safety. However, further clinical trials and exploration of additional therapeutic mechanisms are required to fully understand and evaluate the complete potential of this probiotic mixture.
